# Supplementary material for: A century of weekly notifiable disease incidence data by province in Canada
Source: PLOS Glob Public Health. 2025 Dec 17;5(12):e0005550. doi: 10.1371/journal.pgph.0005550 (PMC12711055; doi:10.1371/journal.pgph.0005550)
Supplement: S1 Appendix — This supporting information details the historical and methodological framework for compiling and preparing Canadian notifiable infectious disease incidence data, and how to access them. It explains the sources and evolution of the Canadian Notifiable Disease Surveillance System (CNDSS), including federal and provincial publications, and describes the process of locating, digitizing, and cleaning these historical records. Data entry and quality control procedures are described, including internal consistency checks across time scales, locations, and disease hierarchies, as well as comparisons among data sources. Finally, the document outlines specific analytical methods for diseases like poliomyelitis and whooping cough. (PDF) [file pgph.0005550.s001.pdf]

# S1 Appendix: Methodological details

## Supporting Information for:
## "A century of weekly notifiable disease incidence data by province in Canada"


David J.D. Earn[1,2], Gabrielle MacKinnon[1,3,4,5], Samara Manzin[1,6], Michael Roswell[1,7,8], Steve Cygu[1,9], Chyunfung Shi[10], Benjamin M. Bolker[1,10], Jonathan Dushoff[2,10], Steven C. Walker[1*]

**1** Department of Mathematics and Statistics, McMaster University, Hamilton, Ontario, Canada
**2** M. G. DeGroote Institute for Infectious Disease Research, McMaster University, Hamilton, Ontario, Canada
**3** Department of Epidemiology, Biostatistics, and Occupational Health, McGill University, Montréal, Québec, Canada
**4** Lady Davis Institute for Medical Research, Jewish General Hospital, Montréal, Québec, Canada
**5** Department of Mathematics and Statistics, McGill University, Montréal, Québec, Canada
**6** Department of Biology, McGill University, Montréal, Québec, Canada
**7** Department of Biology, University of Maryland, College Park, Maryland, United States of America
**8** Department of Entomology, University of Maryland, College Park, Maryland, United States of America
**9** African Population Health and Research Center, Nairobi, Kenya
**10** Department of Biology, McMaster University, Hamilton, Ontario, Canada

* swalk@mcmaster.ca

# Contents


**A Data Sources** **3**

**B Data entry** **6**

**C External links** **7**

**D Data preparation pipelines** **8**

**E Preparing unharmonized CSV files** **8**

**F Preparing harmonized CSV files** **10**

**G Preparing the normalized CSV file** **11**

**H Data provenance** **22**

**I Quality control** **22**

**J Polio methods** **26**

**K Whooping cough methods** **26**

# A Data Sources

In 1924, the Dominion Bureau of Statistics, now Statistics Canada, began collecting communicable disease incidence data.

> The main objectives of this system are to provide a mechanism for monitoring the health of the population by identifying and responding to changes in reporting trends of specific diseases and to provide information that can contribute to the development of health policy and the planning of care, prevention, and control programs. [1]

In 1988 this initiative was transferred to the Canadian Laboratory Centre for Disease Control and persists today under the administration of the Public Health Agency of Canada (PHAC), as the Canadian Notifiable Disease Surveillance System (CNDSS) [2]. Data for the CNDSS are provided by provincial and territorial governments to monitor diseases of public health concern [2]. Prior to the onset of this federal initiative in 1924, several provinces were already collecting such data without reporting it to the federal government.

Our group's search for historical Canadian infectious disease notification data began in 2000. Initially, D. J. D. Earn acquired handwritten weekly counts for Ontario, covering five decades (1939–1989), from the Ontario Ministry of Health (main text Table 1). A significant breakthrough occurred during a visit to the chief medical officer of health in Manitoba, where he discovered a single page of notifications submitted to the Dominion Bureau of Statistics. He photocopied this document and sent it to Statistics Canada, as it was the first evidence that the data tables we were seeking existed.

In 2002 and later, D. J. D. Earn engaged in extensive communications with Statistics Canada via telephone and email. As a result, they provided photocopies of handwritten weekly and monthly notification spreadsheets from 1924–1955 that they had located in their archives. At the time, our resources were insufficient to digitize and clean all of these data. However, we did present analyses of a few disease time series in publications (e.g., [3, 4]). Additionally, we located more published CNDSS data from 1956–2000 in University Libraries and from Statistics Canada. During this historical period, the publication of data transitioned from weekly to monthly and eventually to quarterly. In 2021, with funding from the Canadian Network for Modelling Infectious Diseases (CANMOD), D. J. D. Earn initiated a systematic effort to complete this 2+ decade project to locate, digitize, clean, and distribute all available CNDSS data.

The following paragraphs describe the sources of data, including both federal and provincial government compilations. Throughout the project, D. J. D. Earn, G. MacKinnon, S. Manzin, C. Shi, A. Earn, E. O'Meara, and S. C. Walker contributed to identifying and locating these sources.

**Federal government compilations** The CNDSS data have been published using different titles that have changed over time, many of which are (at the time of writing) difficult or impossible to find using Google without physically going to libraries and archives. Ref. [5]

provides annual communicable disease incidence data by province from 1924 to 1954, but it does not provide a list of the titles used to publish these data. We have been able to find some of these titles, but not all of them. The titles used by Statistics Canada and Health and Welfare Canada to publish CNDSS data changed over time, and we have been able to find some of these titles, but not all of them.

**1924-1959** During this period, the Dominion Bureau of Statistics compiled provincial communicable disease incidence data. Photocopies that we obtained from Statistics Canada are evidence of these compilations. In 1954, the Bureau published annual incidence data from 1924-1952 [5]. This report states that the Dominion Bureau of Statistics began publishing incidence data in 1952 under the title "Summary of Cases of Notifiable Diseases in Canada". This report also states that the Dominion Bureau of Statistics compiled "a restricted monthly release" of incidence data from 1924–1932 and that in 1933 this became a "printed weekly report." The phrase "weekly communicable disease report" was used by Ref. [5], suggesting that this was the name of the "printed weekly report". Again we were unable to find these releases, reports, or publications on the internet, but we have photocopies of data that were presumably used to produce them.

**1959–1974** From 1959-01-24, the Dominion Bureau of Statistics published weekly provincial CNDSS data under the title "Notifiable Diseases – Weekly Summary" until they were renamed Statistics Canada on May 1st 1971. Statistics Canada continued using this name until 1974. Many of these reports from 1959 to 1974 can be found in the Research & Collections Resource Facility at the University of Alberta.

**1975–1976** Statistics Canada and Health and Welfare Canada jointly published weekly provincial CNDSS data using the title "Notifiable Diseases Weekly Summary Provisional Report" for less than one year from 1975-04-26 to 1976-01-03.

**1976–1978** During this period, Health and Welfare Canada published weekly provincial CNDSS data using the previous title "Notifiable Diseases – Weekly Summary".

**1979–1989** During this period, Statistics Canada and Health and Welfare Canada jointly published 4-weekly provincial CNDSS data using the title "Notifiable Diseases Summary". At the time of writing, we were not able to use Google to find a digitized copy of CNDSS data for the first quarter of 1979. However, the Canada Diseases Weekly Reports (CDWR) reproduced CNDSS data using this title starting with the four-weekly period ending 1979-04-21. The CDWR continued to reproduce CNDSS data using this title until 1991, but they did not do so for every four-weekly period. We were able to obtain data for more of these four-weekly periods directly from Statistics Canada, but we were not able to obtain all of them.

**1990–2006** From 1990–1992, Health and Welfare Canada published monthly provincial data under the title "Notifiable Diseases Summary" and continued to publish quarterly provincial data under the same title until the last quarter of 2003 at which time the

Public Health Agency of Canada took over until 2006. Many of these publications are reproduced in the Canada Communicable Disease Report (CCDR).

**2007–present** Unfortunately, we were unable to find any sub-annual and sub-national data source covering all provinces after 2007. Since 2001, the Public Health Agency of Canada (PHAC) has maintained a web portal [2] that provides annual, national incidence.

**Provincial government compilations** Provincial public health agencies have published data directly, and many of these publications predate the federal programme that began in 1924. These sources also varied in the titles under which they published. The following is a non-exhaustive list of titles that have been used by provincial health agencies to publish monthly disease incidence data for their province.

**Ontario, 1903–1905** "The Sanitary Journal of the Provincial Board of Health of Ontario".

**Ontario, 1906–1924** "Annual Report of the Provincial Board of Health".

**Ontario, 1925–1947** "Annual Report of the Department of Health".

**Saskatchewan, 1910** "Annual report of the Bureau of Public Health for the Province of Saskatchewan".

**Saskatchewan, 1921–1922** "Annual report of the Bureau of Public Health of the Province of Saskatchewan".

**Saskatchewan, 1923–1926** "Annual report of the Department of Public Health of the Province of Saskatchewan".

**Saskatchewan, 1927** "Annual report of the Department of Public Health and the Vital Statistics report of the Province of Saskatchewan" .

**Québec, 1915-1922** "Rapport annuel du Conseil supérieur d'hygiène de la province de Québec = Annual report of the Superior Board of Health of the Province of Quebec".

**Québec, 1923-1934** "Rapport annuel du Service provincial d'hygiène de la province de Québec = Annual report of the Provincial Bureau of Health of the Province of Quebec".

**Manitoba, 2004-2017** "Manitoba Monthly Surveillance Unit Report" – available online (at the time of writing) here: `https://www.gov.mb.ca/health/publichealth/surveillance/episummary/archive.html`.

**Provincial and territorial government data requests** In 2021, we began reaching out to provincial and territorial public health agencies for more recent sub-annual data. Although we corresponded via email with all such agencies, only Public Health Ontario and Alberta Health provided us with recent data. Both agencies provided us with weekly data. This work was carried out by G. MacKinnon and S. Manzin, under the supervision of S. C. Walker.

# B Data entry

Data were manually transcribed from scanned reports into standardized Excel spreadsheets following project digitization guidelines:

`https://github.com/canmod/candid/blob/main/digitization_process.md`

The core principle of this document is that digitized spreadsheets should mirror the structure and appearance of the original source documents closely enough for easy comparison, while allowing flexibility when an exact match is impractical. In practice, maintaining consistency across data enterers required more than simply following this principle. Perfect replication was sometimes unrealistic because capturing every small detail demanded more time and effort than available, requiring decisions about which details were worth the effort. In other cases, readability was intentionally improved through small departures from the source, such as alternating row shading. At times, Excel itself could not reproduce the layout of the original–for example, when a number appeared between two table cells–necessitating collective decisions about how to represent such cases in a spreadsheet. The guidelines therefore serve as a shared reference to help data enterers make similar judgments in these situations.

Regular meetings among research assistants were used to resolve ambiguities and update the document. The main team of data enterers included S. Manzin, G. MacKinnon, C. Shi, J. Lin, Q. Zhu, C. Corradi, S. Widrich, J. Maja, and C. Lees. Manzin, MacKinnon, and Shi entered the largest volume of data, with Lees contributing substantial work prior to the start of the systematic digitization effort in 2021. Their work included manual entry from scanned tables into spreadsheets, checking and correcting data entry errors, and scanning hard-copy source documents. Each spreadsheet contains the name of the data enterer in a dedicated cell.

The guideline document is organized around reusable Excel templates that promote consistency across similar sources and formats; these templates contained table structures with row and column headers but no data values, allowing them to serve as standardized starting points for digitizing sources with recurring formats. Some sources were digitized quickly by a single assistant, making separate documentation unnecessary because the resulting spreadsheets themselves defined the format; as a result, not all data sources are represented in the document.

# C External links

Download links to the datasets described in this paper, as well as others from our broader Canadian historical epidemiological data digitization project, can be found at the following web page:

`https://github.com/canmod/iidda/blob/main/README.md`

In addition to download links, which page covers the following additional technical information:

- **Data Dictionary**
- **Data Harmonization**
- **Reproducing IIDDA Datasets**, including:
  - Running natively
  - Running in a Docker container
  - Running interactively
  - Dependency management
  - Requirements
- **Project Structure**, including:
  - Data sources and pipelines (source data and source code)
  - Derived data and tidy datasets
  - Identifiers
  - Metadata
  - Lookup tables
- **Contributions**, including:
  - Contributing source data and pipelines
  - Contributing fixes to data and pipelines
  - Contributing to IIDDA project development

We have also written a small package for reading the data directly into R. An introduction to this package can be found here:

`https://canmod.github.io/iidda-tools/iidda.api/articles/Quickstart`

This `iidda.api` package is part of a suite of packages including tools that are used in data preparation pipelines. The source code for this suite is available here:

`https://github.com/canmod/iidda-tools`

All the code to produce the figures and statistics in this paper are available at:

`https://github.com/canmod/candid`

# D Data preparation pipelines

Each data preparation pipeline followed one of the paths outlined in Fig A. Data sources were provided as hard copies, digitally produced PDF files, or digital spreadsheets. Digital spreadsheets were particularly advantageous because they enabled us to directly script the production of CSV files. When data were not in spreadsheet format, conversion was necessary before scripting could begin. For digitally produced PDF files, we tried to use automated tools like PDFTables (`https://pdftables.com/`) to convert them into spreadsheets. Hard copies, however, always required scanning followed by manual data-entry into spreadsheets. We were unable to find a viable optical character recognition (OCR) approach to avoid manual data-entry. However, in the years since we began this systematic effort to digitize Canadian incidence data there have been tremendous advances in artificial intelligence (AI), and so this situation may have changed. In future work on digitization we plan to replicate parts of this work using OCR, to test it as an efficiency tool in this area.

All CANDID preparation pipelines are available at:

`https://github.com/canmod/iidda/tree/main/pipelines`

The main scripts for producing each of the three datasets (unharmonized, harmonized, and normalized) discussed in this article are available at:

`https://github.com/canmod/iidda/tree/main/pipelines/canmod-compila`
`tions/prep-scripts`

These scripts depend on outputs produced by other pipelines that generate data from specific sources. Instructions on how to reproduce all of these outputs are provided in the `README.md` file on this GitHub repository:

`https://github.com/canmod/iidda`

The data preparation pipelines were written by G. MacKinnon, S. Manzin, F. Jin, and S. C. Walker. These individuals, in addition to S. Lee, R. Jin, M. Roswell, and J. Freeman, also wrote software packages that were used in these pipelines. The reason for separating pipelines from packages is that pipelines are specific to this project, whereas packages are designed to be reusable in other digitization projects. The packages used in these pipelines are available at:

`https://github.com/canmod/iidda-tools`

# E Preparing unharmonized CSV files

We developed one open-source R script for each spreadsheet that converts it into a tidy CSV file [6]. These R scripts used the `unpivotr` package [7] to convert the wide-format data used in historical documents to long-format data that make it easier to manipulate using standard tools [6]. Long-format data also make it easier to combine data from different sources into

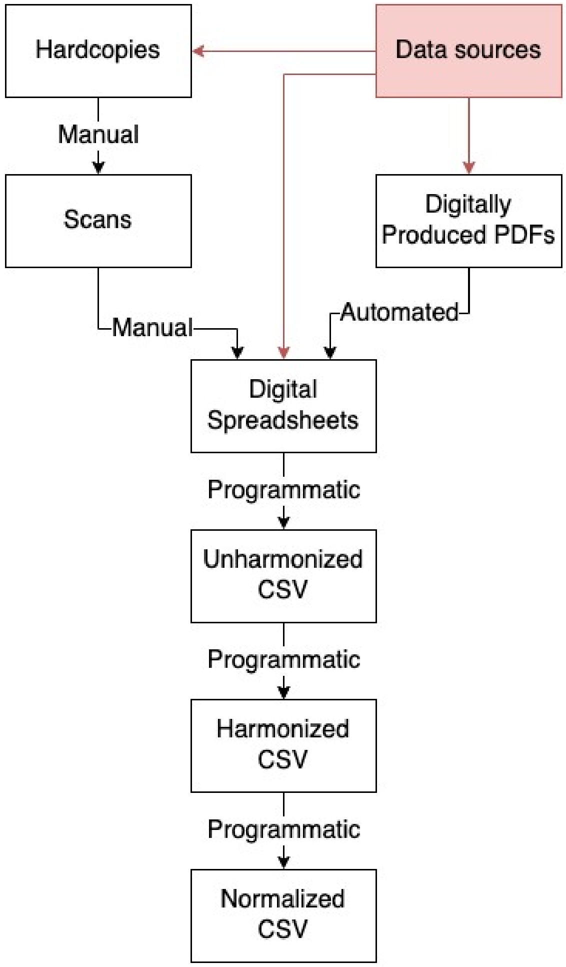


Fig A: Data preparation pipeline overview. All products begin with data sources (red), which provide hard copies, digital spreadsheets, or digitally produced PDFs. Processing steps are classified as manual (e.g., data-entry), automated (e.g., PDF table extraction tools), or programmatic (e.g., customized R script).

a single data set, by ensuring they each consist of fields from the same standardized data dictionary. All of the resulting CSV files have been collected into a single file that we label *unharmonized* because it contains unharmonized historical disease and place names.

These unharmonized data also include information on why certain incidence values are not available, when we could determine the reason from the sources. In the column containing numbers of cases, `cases_this_period`, we allowed the following types of values:

- Non-missing numeric case numbers (non-negative integers).
- One of the following strings explaining why the case numbers are missing[1]:
  - The phrase '`Not available`', for unknown reasons.
  - The phrase '`Not reported`', for unknown reasons.
  - The phrase '`Not reportable`', presumably indicating that the jurisdiction was not required to report these numbers.
  - The word '`Missing`', typically indicating missing pages in the middle of a multi-page table.
  - The word '`Unclear`', meaning that the value is missing from CANDID because it is not legible.
  - The word '`Unclear`', with a special string format,

    `{guess_1} - {guess_2} - ... - {guess_n} (unclear)`

    (e.g., '`36-23-59 (unclear)`'), meaning that the value is missing because the number is difficult to read but we have one or more guesses. In the harmonized datasets we use the first (i.e., best) guess (see the section on Preparing harmonized CSV files).
  - Phrases of the format '`Wrong but clear total in this cell is {value}`', meaning that this cell should contain a marginal total (e.g., annual total), and that the value is clearly written, but is not the correct total obtained by summing up the component values (e.g., weekly counts).

This list of values for recording missing and unclear data is also described in our living document of data entry processes, introduced in the Data entry section.

# F Preparing harmonized CSV files

We developed one open-source R script for each data source that joins the unharmonized data with harmonized disease and place names. This was achieved by creating lookup tables containing all historical names, and then adding columns for harmonized names. Links to these lookup tables are available at:

[1]Typically these strings were taken as is from the source, but in many instances before 1924 we had to make an educated guess about the reason why particular records were missing.

`https://github.com/canmod/iidda/blob/main/README.md#canmod-digitization-project`

Because sources sometimes reported diseases hierarchically, our harmonized disease names were provided in two columns: `disease`, giving the name of the disease being reported, and `nesting_disease`, optionally giving the name of another disease within which the `disease` is nested. We refer to the values in these `disease` and `nesting_disease` columns collectively as ***disease names***. These disease names are organized into hierarchies, such that most disease names are nested within another disease name (e.g., hepatitis-A is nested within hepatitis-A-B). We refer to disease names that are not nested within any other disease name as ***basal diseases***. These basal diseases are plotted in Figures 4-6 of the main text. For a given combination of location and time period, all disease names at or below a specific basal disease in the hierarchy are referred to as its ***sub-diseases***.

In addition to joining harmonized names, the harmonization scripts also apply the following changes:

- Apply fixes to dates and locations that were obviously entered incorrectly in the original source documents (e.g., one whooping cough record from 1943 was for a week ending on a Sunday, while all other data from the same source were for weeks ending on a Saturday).
- Aggregate data that were stratified by age or city.
- Replace alternative characters for reporting zero cases with a literal `0` (e.g., often a dash was used).
- Remove records containing missing values or text strings indicating the type of missing value (e.g., '`Unclear`', '`Missing`').

These fixes add convenience at the expense of removing information contained in the original source, but this information remains accessible in the unharmonized data.

We collected all of the resulting harmonized CSV files into a single file that we label *harmonized*.

# G Preparing the normalized CSV file

We developed an open-source R script to remove overlapping data and to add data that are implied but not explicitly provided by the original source documents. Our goal was to ensure that each apparently reported case is represented by a single incidence value in the resulting CSV file. We refer to the resulting file as *normalized* aligning with the concept that normalized databases represent each 'fact' only once [6].

There are five sources of overlap that could cause cases to be counted more than once:

- Locations (e.g., provincial data being reported along with national data)

- Data sources (e.g., weekly data for Ontario between 1939–1978 being reported by both Statistics Canada and the Ontario Ministry of Health)
- Time periods (e.g., weekly data being reported along with monthly data)
- Disease hierarchies (e.g., polio with and without paralysis being reported along with total polio)
- Mixtures of the previous sources of overlap (e.g., some provinces have only monthly data, while others have only weekly data.)

In addition to removing overlapping historical records, we also add records that are implied by the information in the sources. There are two types of implied information in the harmonized data that we have made explicit in the normalized data:

- Unaccounted cases that are detected when the reported number of cases for a disease is greater than the total over its sub-diseases.
- Missing data at finer time-scales (e.g., weekly, monthly) can be assumed to be zero if zeros are reported at a coarser time-scale (e.g., yearly data) that temporally bound the finer scale.

The CANDID archive curates data from a wide variety of sources (main text Table 1) and diseases, each with different biases and quality issues. It is therefore impossible to produce a perfectly normalized dataset, nor is it our goal to do so here. Instead we aim (1) to make reasonable choices so that the analyses in this paper respect basic principles of consistency (e.g., each case is counted at most once), (2) to set up a data processing pipeline that allows sustained work to improve the quality of the normalization process, and (3) to make the complete and un-normalized data easy to access so that others with expertise in a particular area can make improved normalization choices.

In the remainder of this section, we describe the normalization steps that we take to produce our posted normalized files. Users are free to modify these steps by modifying the scripts and other files these scripts depend on (see the Data provenance section for information on how to find these scripts and files).

**Add unaccounted cases:** Sometimes the total for a `nesting_disease` is reported along with some, but not all, of its sub-diseases. In these instances, after having ruled out other known data quality issues, we produced records with incidence counts given by the associated reported total minus the sum of the reported sub-diseases. These incidence values can be identified in the normalized dataset by a value in the `disease` column of the form `nesting-disease_unaccounted`, and with `derived-unaccounted` in the `record_origin` column. These sums were computed by grouping by time-period, province, data source ID, and `nesting_disease`.

**Join population data:** The estimated provincial population for each incidence value was joined to the normalized dataset. As a result, population numbers are repeated in the dataset because incidence values for different diseases are linked to the same population size within a specific period and province. While this repetition technically violates our normalization principle, the added convenience justifies this step. The `population` column provides linearly interpolated estimates of the intercensal populations for each province at the mid-point of each period, using census-derived data from [8–10].

**Resolving overlapping locations:** Filtering out all data for the entire country easily solves this source of overlap.

**Compute implied zeros:** In some instances, there are zeros reported at a coarse timescale (i.e., for a year), but the data at a finer timescale (weekly/monthly) for the same disease and location is empty or not available. We replaced weekly data that were missing and/or not available in national data sources with zeros when they were implied by a zero at a coarser timescale for the same disease and location. These incidence values can be identified in the normalized dataset by a value of `derived-implied-zeros` in the `record_origin` column. These implied zeros were given lower priority than other weekly data when resolving overlap, as we describe next.

**Resolving overlapping data sources and time-scales:** We generally prioritize national data sources that report for all provinces (e.g., Statistics Canada) over provincial data sources that report for a single province (e.g., Saskatchewan Bureau of Public Health). We always prioritize finer time-scales (e.g., weekly) over coarser ones (e.g., quarterly). For example, if monthly data from a national source overlaps with weekly data from a provincial source, we will choose the weekly provincial data.

We handle data source and time-scale overlap sequentially, starting with an empty dataset and adding records from a dataset produced by applying the previous normalization processes to the harmonized data. At each step in this sequence, we consider new candidate records and only add those that do not overlap temporally with the existing ones. Being added first therefore indicates a higher priority:

1. All weekly data from national sources.
2. Non-overlapping weekly data from provincial sources.
3. Non-overlapping two-weekly data from national sources.
4. Non-overlapping two-weekly data from provincial sources.
5. Non-overlapping weekly implied zeros from national sources.
6. Non-overlapping monthly data from national sources.
7. Non-overlapping monthly data from provincial sources.

8. Non-overlapping quarterly data from national sources.

For any time period and province, we have at most two data sources: one from a federal organization (e.g., Statistics Canada) and one from a provincial organization (e.g., Saskatchewan Bureau of Public Health). To address such overlap when it occurs, we prefer national sources to those from provincial sources. This choice has the advantage of being easy to apply and also has a better chance of producing provincial data streams that are comparable because we can inherit the choices that the federal organization made when publishing data from different provinces.

If the two sources produced identical results then this choice would be irrelevant. Although there are periods for which national and provincial sources reported identical counts, this is not typically the case. Fig B gives an example comparing 37 years of weekly whooping cough data in Ontario as reported by Statistics Canada and the Ontario Ministry of Health. This figure shows that until 1970 the two agencies were reporting virtually identical numbers, with the occasional deviation. In contrast, there are deviations consistently from 1970 to 1977, although the qualitative pattern is still similar.

**Resolve overlap caused by disease hierarchies:** To address how this type of overlap is addressed, it is necessary to further define terminology related to disease hierarchies, building on the concepts introduced in the section on Preparing harmonized CSV files. The ***global hierarchy*** of a basal disease includes all sub-diseases that appear at least once in the harmonized dataset, while the ***local hierarchy*** is specific to a particular location and time period. Some of these global hierarchies are simple (e.g., whooping cough has no sub-diseases at all) whereas others are complex (e.g., meningitis has 37 sub-diseases in the global hierarchy) with local hierarchies changing over time.

We will dig into the meningitis hierarchy a little to give a sense of the complexity. The harmonized dataset contains 33 different local hierarchies of meningitis. The website for the paper has one figure for each of these local hierarchies at this URL: `https://github.com/canmod/candid/tree/main/output/disease-hierarchies`. Here we plot and discuss four of them (Figs C to F). Each of these figures give the meningitis global hierarchy, highlighting in blue the sub-diseases for a particular local-hierarchy (with all other diseases in red). Disease names starting with "ex" indicate counts that exclude certain disease types. Full disease names are constructed by concatenating node names along the hierarchy with dashes (e.g., meningitis-bacterial-haemophilus-influenza), although some names are abbreviated in the figures to save space. Here we summarize the four local hierarchies illustrated in these figures:

- From 1921 to 1967 Statistics Canada reported a total meningitis count without any sub-diseases (Fig C). The sources give no indication of what kind of meningitis is being reported, possibly because it was not known.

- From 1969–1978 only viral meningitis was reported and this was stratified by coxsackie, echo, and virus-unspecified (Fig D). Even if totals for meningitis-viral or meningitis

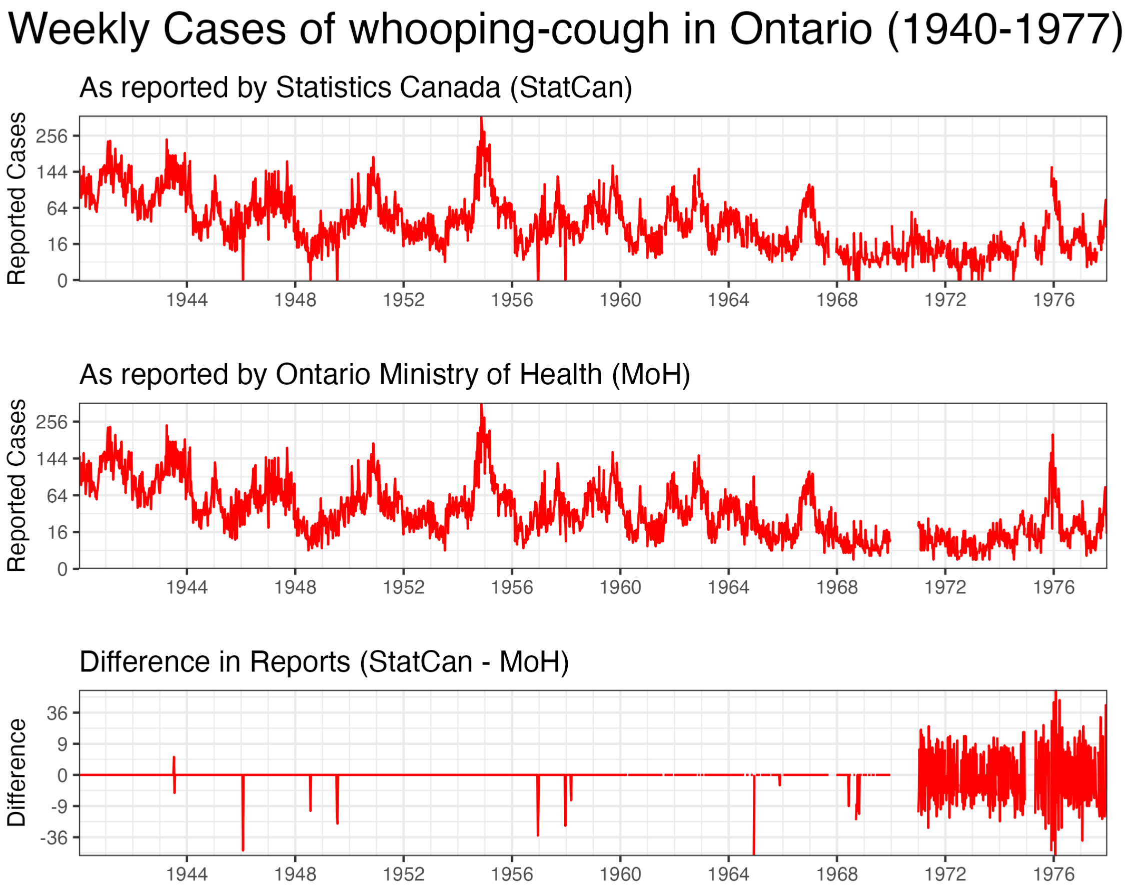


Fig B: Comparing reported Ontario whooping cough incidence from Statistics Canada with the Ontario Ministry of Health. The difference between the two sources is given on the bottom panel. Additional comparisons among similar data from different agencies can be found on the website for the paper: `https://github.com/canmod/candid/tree/main/output/agency-comparisons`

were given in these sources, they were excluded from the normalized data to avoid overlap.

- From 1979 to 1985 the collection of sub-diseases changed completely to report only meningitis associated with encephalitis (both viral and bacterial, Fig E).
- From 2004 to 2007, Manitoba Health reported a complex collection of sub-diseases (e.g., Fig F). Even in this complex case, there is no overlap in observed diseases in the normalized data.

These examples show how we prevent overlap in disease hierarchies. For each basal disease, we keep only the most detailed sub-diseases that were actually reported for a given place and time. Any intermediate or higher-level totals are removed. In effect, all retained sub-diseases are treated as children of the basal disease, ensuring that every case is counted exactly once. For example, Statistics Canada's 1969–1978 meningitis data (Fig D) included counts for coxsackie, echo, and virus-unspecified meningitis, but also listed a total for viral meningitis. We discarded this total during normalization, so the viral node appears in red to indicate that it is not observed in the normalized data.

For example, the stratification of meningitis illustrated in Fig D came from a data source (Statistics Canada) that also reported a total for viral meningitis (historically called aseptic meningitis), but this total was removed in the normalization process and so the viral node is coloured red to indicate that this sub-disease cannot be observed in the normalized data. As another example, Public Health Ontario reported acute and chronic hepatitis B (Fig G, top), while Statistics Canada and some provinces reported only a single hepatitis B total (Fig G, bottom). Our approach keeps whichever sub-diseases were actually reported locally and removes overlapping intermediate totals.

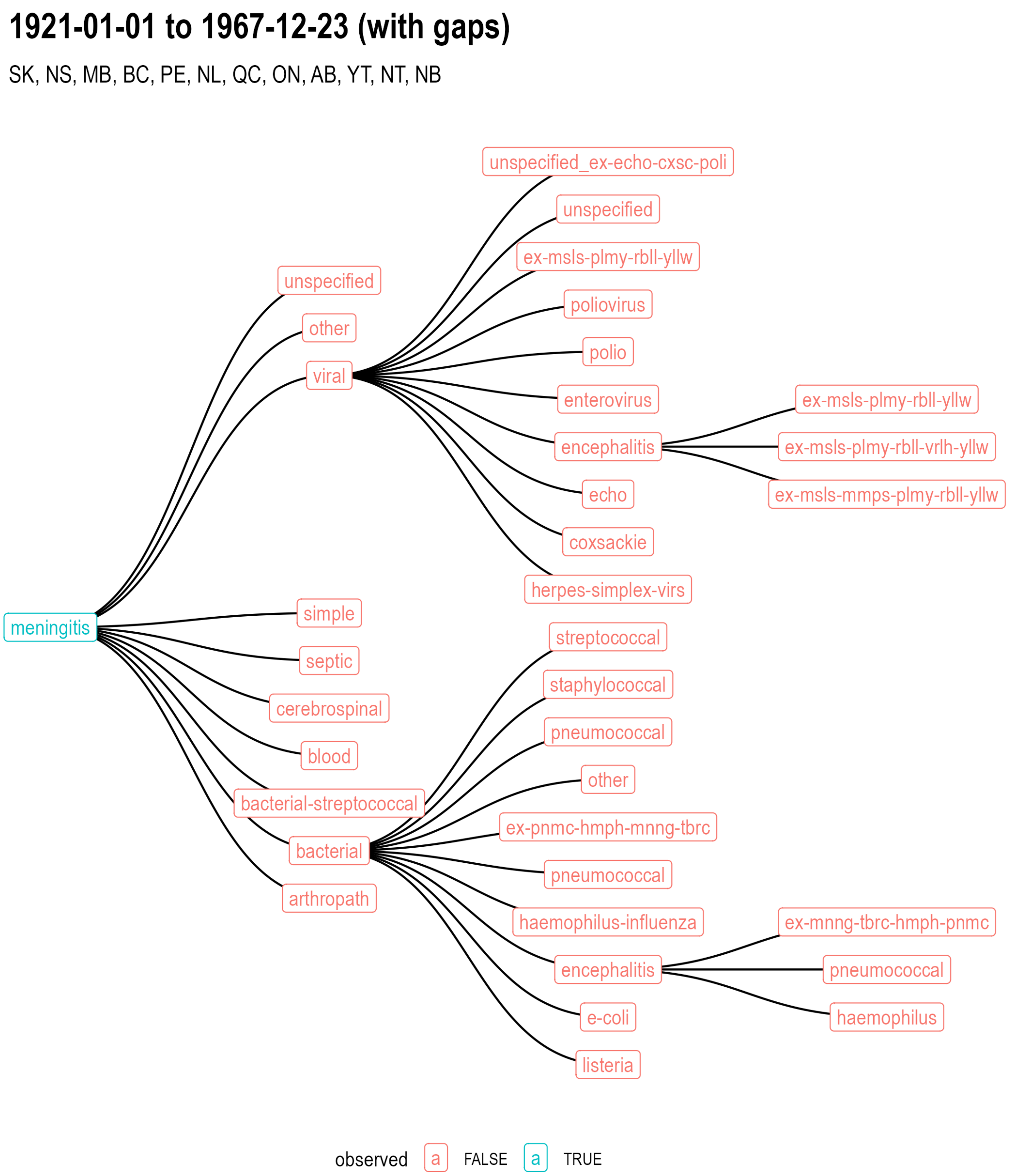


Fig C: Global meningitis disease hierarchy highlighting in blue a particular local hierarchy of reported sub-diseases between 1921 and 1967. In this local hierarchy, the only reported sub-disease is the basal disease itself.

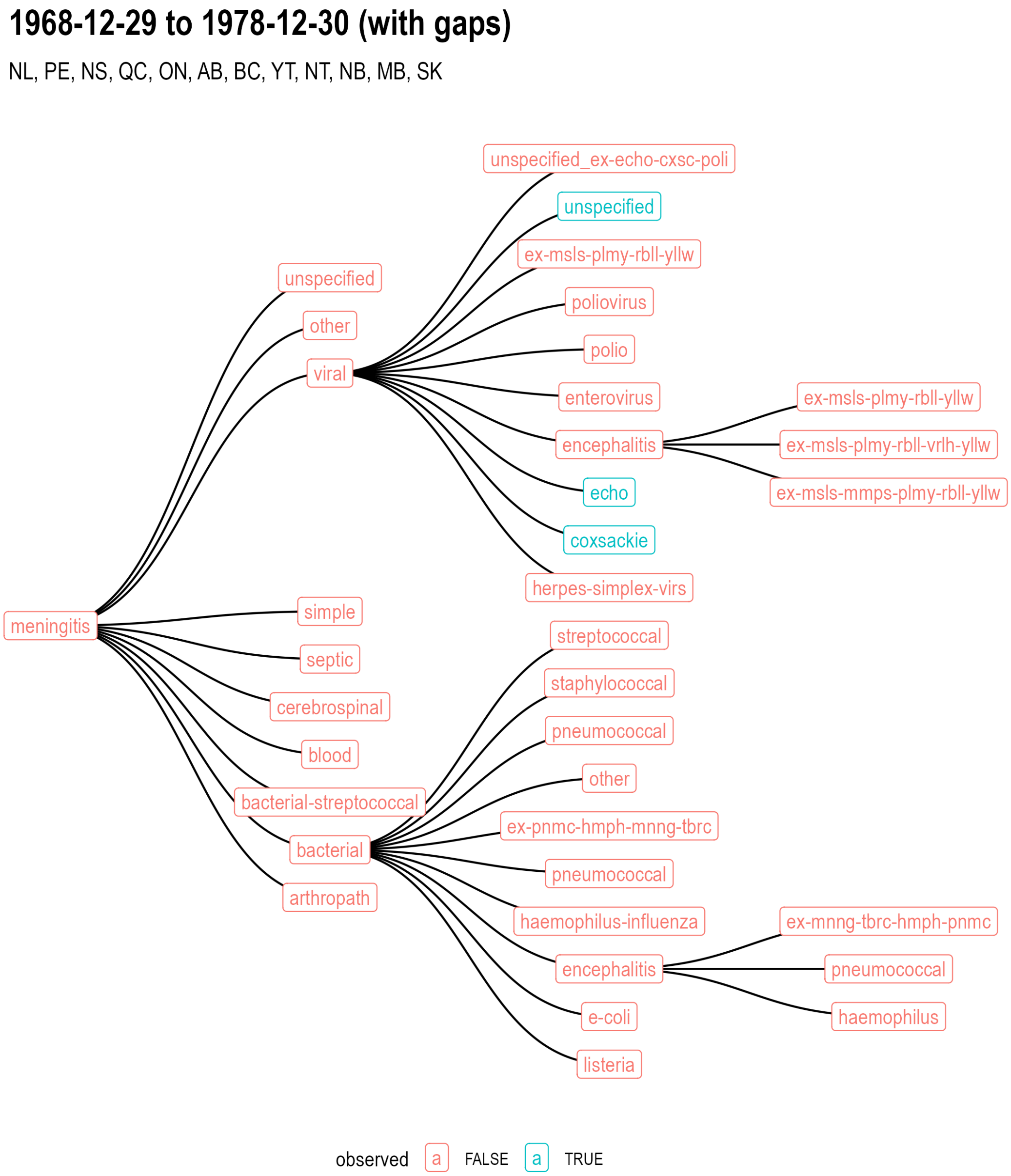


Fig D: Global meningitis disease hierarchy highlighting in blue a particular local hierarchy of reported sub-diseases between 1968 and 1978.

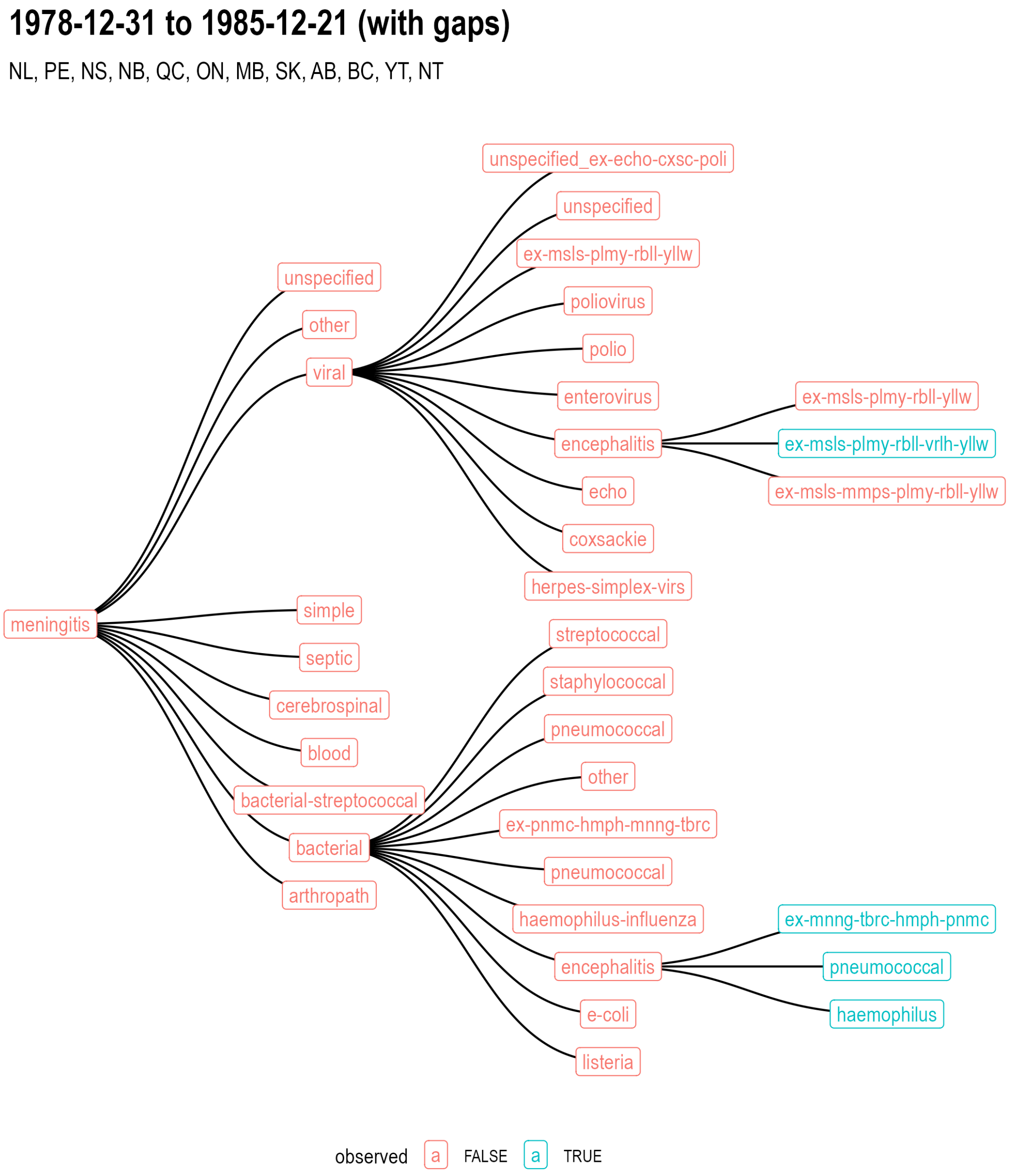


Fig E: Global meningitis disease hierarchy highlighting in blue a particular local hierarchy of reported sub-diseases between 1979 and 1985.

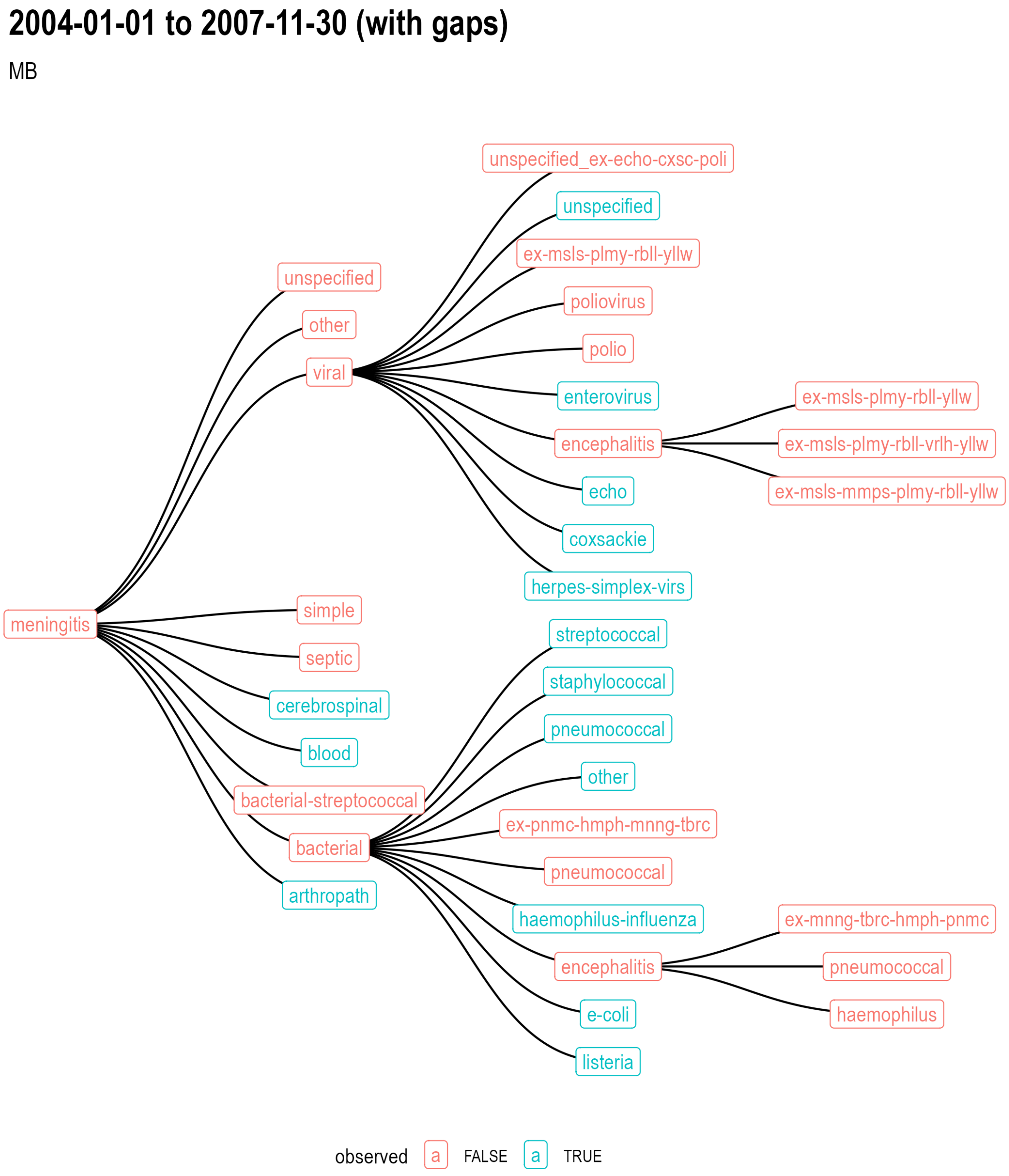


Fig F: Global meningitis disease hierarchy highlighting in blue the sub-diseases reported by Manitoba Health in the date ranges given.

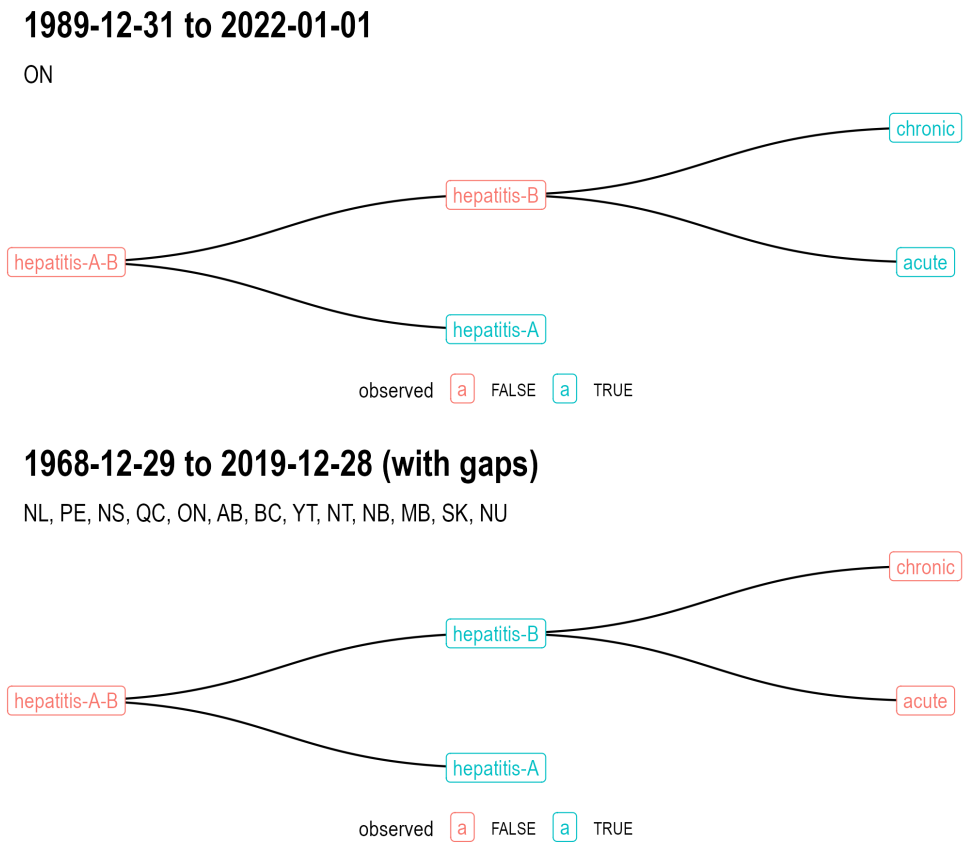


Fig G: Global hepatitis A and B disease hierarchy, with two panels highlighting different local hierarchies in blue. In the top panel hepatitis B is stratified by acute and chronic sub-diseases, but in the bottom panel hepatitis B is not stratified. See Figs C to F for interpretation details. All hierarchies for hepatitis can be found on the website for the paper: `https://github.com/canmod/candid/tree/main/output/disease-hierarchies`

# H Data provenance

The harmonized and normalized data both contain the following columns with unique identifiers to resources used to produce each record:

- `original_dataset_id`: Uniquely identifies the unharmonized dataset containing each record.
- `digitization_id`: Uniquely identifies the digitization (typically an Excel file) containing the data for each record.
- `scan_id`: Uniquely identifies the scan of the original document containing each record.

Records that were implied from associated data (see the section on Preparing the normalized CSV file), as opposed to explicitly reported, do not have entries in the first two of these columns. Data that we received in digital form typically do not have a scan associated with them, and so such records do not have a `scan_id`.

Information on the above identifiers can be found at:

`https://github.com/canmod/iidda/blob/main/README.md#identifiers`

The following vignette describes how to investigate the provenance of a record using these identifiers:

`https://canmod.github.io/iidda-tools/iidda.api/articles/Provenance`

Metadata for the unharmonized, harmonized, and normalized versions of our prepared data are available at:

`https://github.com/canmod/iidda/blob/main/README.md#canmod-digitization-project`

These metadata contain links to all of the resources (R scripts, Excel/CSV files, and PDFs) used to produce the datasets.

# I Quality control

We performed two types of quality control checks: marginal total cross checks and comparisons with the PHAC portal. This work was performed by S. C. Walker, G. MacKinnon, S. Manzin, S. Cygu, and Q. Zhu. After describing these two types of checks, we describe how the results of these checks were used to improve the quality of the data by fixing data-entry errors and improving our normalization process.

**Marginal total cross checks:** Using marginal totals from the comprehensive dataset, we performed cross checks over the three available stratifications of data: time scales, locations, and diseases. The analysis involved several comparisons at different levels of data aggregation.

For time-scales, sub-annual records, such as weekly, monthly, or quarterly data, were aggregated to the annual scale and compared to the available annual records. When multiple sub-annual time scales were present, their annual sums were also compared to one another.

For locations, sub-national data were aggregated to the national level, and this sum was compared with the reported national total. Both the national and sub-national totals were aggregated to yearly scales for comparison with records in the PHAC portal.

For diseases, sub-class totals were aggregated and compared to the overall disease totals. In cases where the sum of sub-diseases was less than the reported disease total, the difference was labeled as `disease_unaccounted` and included in the normalized data as a derived entry with a `record-origin` of `derived-unaccounted-cases`.

For each cross check, we put all records with discrepancies into a CSV file with provenance information for finding the original scans and excel files to more easily fix potential errors. The scripts for producing these CSV files can be found at:

> `https://github.com/canmod/iidda/tree/main/pipelines/canmod-cross-checks/prep-scripts`

A link to download the current state of these CSV files, along with the data themselves, is located at:

> `https://github.com/canmod/iidda/blob/main/README.md#canmod-digitization-project`

We plan to continue addressing these potential issues, but if you use the archive, please check the discrepancy files to see if any data of interest is flagged. Given that our pipelines and tools are open, users are encouraged to fix issues and submit pull requests to our GitHub repository:

> `https://github.com/canmod/iidda`

The following list summarizes the current status of each cross-check:

- Time-scale cross checks
  - Total number of year-location-disease combinations with discrepancies: 365
  - Percentage of these combinations without these discrepancies: >99%
  - Percentage of these discrepancies that are from handwritten data: 100%
- Location cross checks
  - Total number of period-disease pairs with discrepancies: 2,036

    - Percentage of these pairs without these discrepancies: >99%
    - Percentage of these discrepancies that are from handwritten data: 63%

- Disease cross checks
    - Total number of period-location pairs with discrepancies: 183
    - Percentage of these pairs without these discrepancies: >99%
    - Percentage of these discrepancies that are from handwritten data: 88%

It is difficult to combine this information into an estimate of the overall proportion of the harmonized dataset that is error free, because the above estimates are for different stratifications of the data. We can expect errors to yield discrepancies in more than one cross check. For example, a single data-entry error in a sub-disease, province, and week could trigger a discrepancy in all three cross checks if the disease, national, and annual data were reported as well. Not all of these discrepancies represent our data-entry errors because sometimes the original sources are inconsistent or unclear (especially in the handwritten data). Sources also varied in the quality of their marginal totals, and so we removed these totals from our cross checks. But overall, given that the percentages of the combinations of factors that are free of known discrepancies are all greater than 99%, we do not expect that many more data-entry errors remain.

**Comparing with the PHAC portal:** We also compared the national and yearly data on the PHAC portal (`https://diseases.canada.ca/notifiable`) with aggregated national and yearly totals in CANDID for what we believe are the same diseases. We made these comparisons visually using line plots. Fig H gives an example using chickenpox.

We do not expect the data sources to match exactly, for a variety of reasons. Before 1924 and after 2000 all of the data come from provincial data sources, and so our spatial coverage is limited at these times, whereas the PHAC portal typically reports using data from more provinces after 1924 (though not always, as is evident in the bottom panel of Fig H). CANDID historical source documents presumably included the best numbers at the time. PHAC may have updated these numbers to account for data quality corrections or changes in criteria for determining whether a case qualifies as a specific disease. Discrepancies could also occur because the sub-diseases included in a particular disease change over time.

**Fixing data:** These data quality checks have allowed us to correct errors, and to continue to do so, including data-entry typos (e.g., if 500 cases should have been 50), fixing bugs in preparation scripts, and rethinking the interpretation of data source organization. For instance, the difference between zero incidence and missing incidence was not clear in some of the handwritten data (main text Fig 2). Additionally, we needed to change how we aggregated sub-diseases to be comparable with aggregations on the PHAC portal.

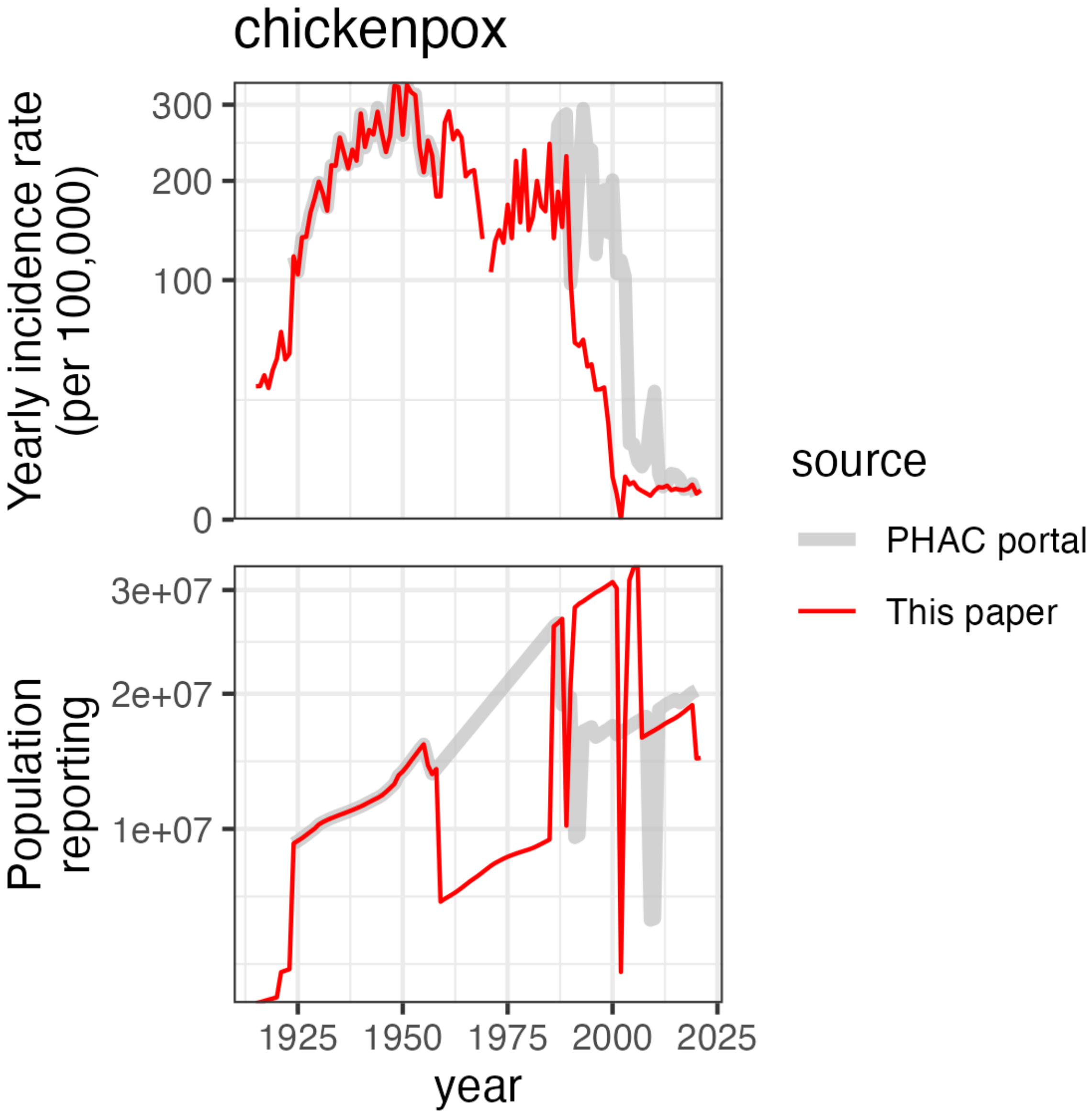


Fig H: Chickenpox as an example of a comparison between our archive and the PHAC portal. The top panel gives yearly incidence from CANDID in red and the PHAC portal in grey. The bottom panel gives the total population of all reporting provinces for each source. Jumps in this bottom panel are caused by provinces being added or dropped from each source. For chickenpox the reported incidence is very similar in both sources during times when the reporting population is identical. Comparisons for all diseases that are shared by both datasets can be found on the website for the paper: `https://github.com/canmod/candid/tree/main/output/phac-portal-comparisons`.

# J Polio methods

We analyzed weekly provincial polio data. For each province and week, we calculated the weekly incidence rate by multiplying the number of new cases by 100,000 and dividing by the interpolated population for that week. To estimate the national incidence rate, we summed the provincial cases and divided by the population of provinces with available data, then multiplied by 100,000. Vertical lines in the plot (main text Fig 7) indicate the week with the highest national incidence in a given polio year, which we defined as the 52-week period centered around epidemiological week 34, the typical yearly peak for polio. We plotted peaks only for years with more than 20 total cases reported in the country.

We identified the peak polio week for each combination of province and polio year. We defined the peak as the week in the polio year with the highest incidence. To ensure sufficient data for estimating a clear peak, we excluded combinations with sparse or limited case counts ($\leq 5$ weeks of non-zero incidence or $\leq 20$ total cases). Fig I shows the distribution of peak weeks by province and highlights that most provinces in most years peaked between August and October.

# K Whooping cough methods

We estimated the annual incidence rate in each of the six geographic regions of Canada, and Canada as a whole, as follows. We first computed the average daily number of whooping cough cases for each province or territory by summing the reported cases over all available time periods within a year and dividing by the total number of observed days. We then estimated the total annual cases for each province by scaling this daily rate by the total number of days in the year, accounting for any missing data. Summing these estimates across the provinces within each geographical region gave us the total estimated cases per region. Finally, we calculated the incidence rate per 100,000 individuals by dividing the regional total estimated cases by the total regional population and scaling appropriately. These sums and averages were not contaminated by double counting, as each reported or implied case is represented only once in the normalized dataset (see the section on Preparing the normalized CSV file). However, our method could be affected by within-year variation in incidence rates that did not average out over the sample of available time periods.

Details for these calculations are as follows:

- Let $x_{ij}$ be the number of new whooping cough cases reported during time period $i$ (e.g., week, month, or quarter) within province or territory $j$ of Canada.
- Let $n_{ij}$ be the number of days in time period $i$ within province or territory $j$.
- Let $\Omega_k$ be the set of all time periods $i$ within year $k$. Note that $\Omega_k$ may not include all possible periods if data are missing for some weeks, months, or quarters.
- Let $\Psi_l$ be the set of provinces and territories $j$ that are contained within geographical region $l$ of Canada (e.g., Atlantic, Quebec, Ontario, Prairies, British Columbia,

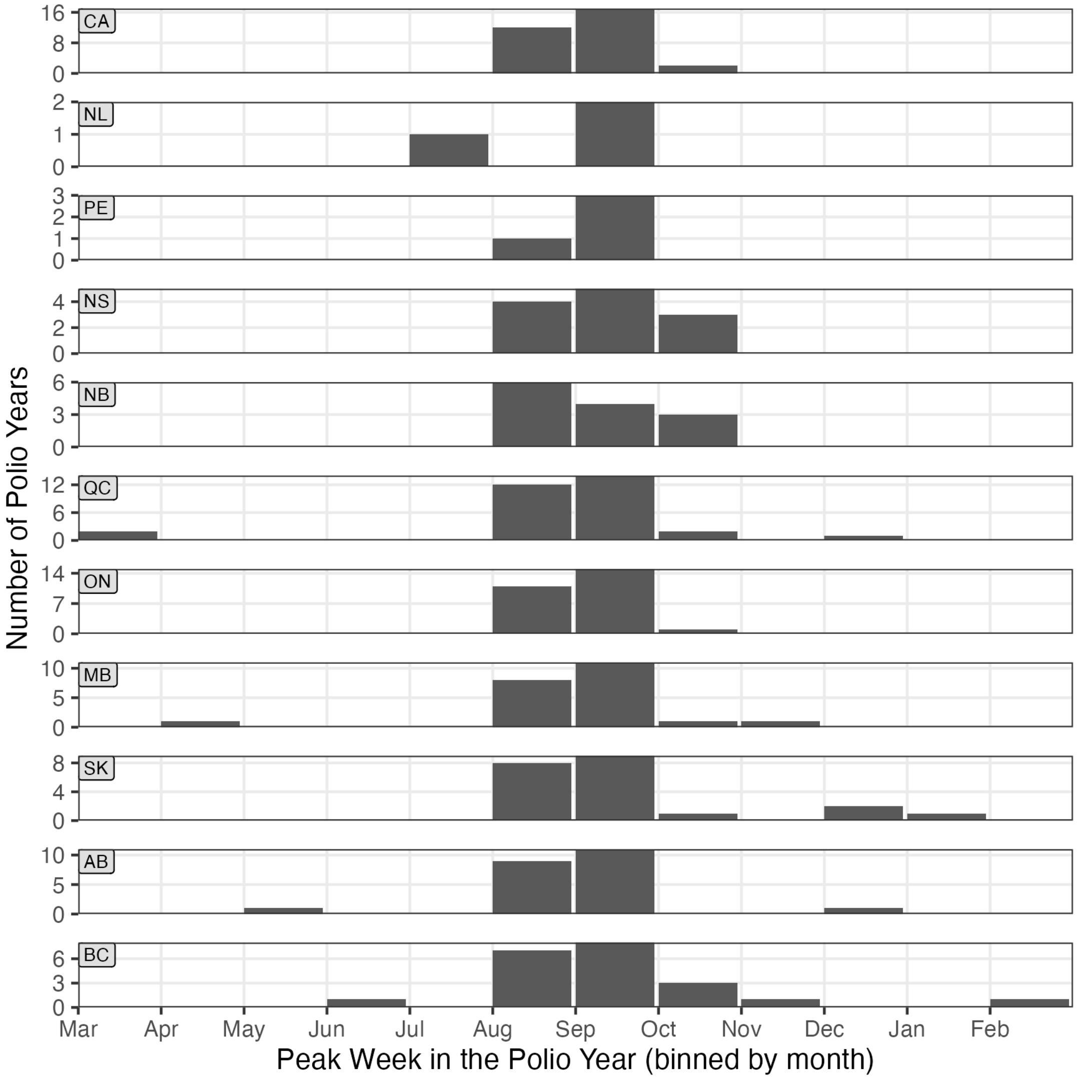


Fig I: Histograms of the distribution of the peak week binned by month. Each panel gives the distribution for Canada (top panel) and each province.

Territories).

- Let $N_k$ be the total number of days in year $k$ (either 365 or 366).
- Let $p_{kj}$ be the population of province or territory $j$ during year $k$.

1. **Average Daily Cases in Year $k$ and Province $j$:**

$$\text{Average Daily Cases}_{kj} = \frac{\sum_{i \in \Omega_k} x_{ij}}{\sum_{i \in \Omega_k} n_{ij}}$$

2. **Estimated Total Annual Cases in year $k$ in Province $j$:**

$$\text{Estimated Annual Cases}_{kj} = N_k \times \left( \frac{\sum_{i \in \Omega_k} x_{ij}}{\sum_{i \in \Omega_k} n_{ij}} \right)$$

3. **Total Estimated Cases in Year $k$ and Region $l$:**

   Sum the estimated annual cases over all provinces and territories $j$ within region $l$:

$$\text{Total Estimated Cases}_{kl} = N_k \times \sum_{j \in \Psi_l} \left( \frac{\sum_{i \in \Omega_k} x_{ij}}{\sum_{i \in \Omega_k} n_{ij}} \right)$$

4. **Incidence Rate per 100,000 Individuals in Year $k$ and Region $l$:**

   Calculate the incidence rate by dividing the total estimated cases by the total population of the region and scaling by 100,000:

$$\text{Incidence Rate}_{kl} = 100,000 \times N_k \times \frac{\sum_{j \in \Psi_l} \left( \frac{\sum_{i \in \Omega_k} x_{ij}}{\sum_{i \in \Omega_k} n_{ij}} \right)}{\sum_{j \in \Psi_l} p_{kj}}$$

# References


[1] Sockett PN, Garnett MJ, Scott C, et al. Communicable disease surveillance: Notification of infectious diseases in Canada. Canadian Journal of Infectious Diseases and Medical Microbiology. 1996;7:293-5.

[2] Totten S, Medaglia A, McDermott S. Updates to Canadian Notifiable Disease Surveillance System. Canada Communicable Disease Report. 2019;45(10):257-61. Available from: `https://doi.org/10.14745/ccdr.v45i10a02`.

[3] Bauch CT, Earn DJD. Transients and attractors in epidemics. Proceedings of the Royal Society of London, Series B. 2003;270(1524):1573-8.

[4] Hooker G, Ellner SP, De Vargas Roditi L, Earn DJD. Parameterizing state-space models for infectious disease dynamics by generalized profiling: measles in Ontario. Journal of the Royal Society of London, Interface. 2011;8(60):961-74.

[5] Canada Dominion Bureau of Statistics Health and Welfare Division Public Health Section. Summary of Notifiable Diseases, 1924–1952 = Sommaire de maladies à déclaration obligatoire, 1924-1952. Ottawa: Queen's Printer; 1954. Available from: https://publications.gc.ca/site/eng/9.846794/publication.html.

[6] Wickham H. Tidy data. The Journal of Statistical Software. 2014;59. Available from: http://www.jstatsoft.org/v59/i10/.

[7] Garmonsway D. unpivotr: Unpivot Complex and Irregular Data Layouts; 2023. R package version 0.6.3. Available from: https://CRAN.R-project.org/package=unpivotr.

[8] Dominion Bureau of Statistics, Canada. Sixth Census of Canada, 1921. vol. 2. Dominion Bureau of Statistics; 1925. Available from: https://publications.gc.ca/pub?id=9.830550&sl=0.

[9] Statistics Canada. Population-1921-1971-Revised Annual Estimates of Population, by Sex and Age Group, Canada and the Provinces. Statistics Canada; 1973. Available from: https://publications.gc.ca/pub?id=9.817507&sl=0.

[10] Statistics Canada. Table 17-10-0005-01 Population estimates on July 1, by age and gender; 2021. Accessed: 2022-02-16. Available from: https://doi.org/10.25318/1710000501-eng.
