# Supplementary material for: A century of weekly notifiable disease incidence data by province in Canada
Source: PLOS Glob Public Health. 2025 Dec 17;5(12):e0005550. doi: 10.1371/journal.pgph.0005550 (PMC12711055; doi:10.1371/journal.pgph.0005550)
Supplement: Attachment — Submitted filename: submitted-response-to-review.pdf [file pgph.0005550.s002.pdf]

Thank you for your thoughtful and constructive review. Below, we provide a detailed point-by-point response to each of your comments. We have included a marked up PDF file highlighting the changes that we discuss below.

> **Reviewer**: *The epidemiological analysis lacks novelty, as the seasonal nature of polio is well-established.*

We have shortened and clarified the analysis (in the 1st paragraph of the Examples section) . We agree that much is well-understood about polio seasonality, but also feel that our analysis adds value. In particular, it is of interest that we see later epidemics in Canada than in the US, but no evidence of difference between colder and less-cold provinces within Canada. We have clarified these interpretations in the 3rd paragraph of the Explanation section.

> **Reviewer**: *The paper is excessively long with sections that are difficult to comprehend, reducing its overall clarity and impact.*

We have comprehensively edited the main text for clarity and brevity. The current main text is 2458 words. The appendix in the Supporting Information is still fairly long. We wanted to err on the side of providing technical details for people who want to reproduce analyses. We have also clarified that the appendix serves as Supporting Information by following PLoS style: (1) including a brief description of the Supporting Information before the Acknowledgements; (2) using more explicit in-text references to Supporting Information (e.g., "Fig S9 in S1 Appendix"); (3) providing clickable links to the description of "S1 Appendix" and to specific elements within it, such as "Fig S9," for the convenience of reviewers.

> **Reviewer**: *There is insufficient discussion of NOID (Notifiable Disease) statutes across Canadian is provinces and their evolution over time.*

In the Introduction (in the 2nd paragraph) we now point to the Supporting Information (pages S1-S4), which now systematically documents where and how Canadian infectious disease surveillance data have been published, identifying key federal and provincial sources, publication titles, and time ranges. By detailing the history of data publication, describing specific archival discoveries, and listing concrete sources—many not findable online—it serves as a practical guide for researchers seeking additional data. In doing so, it directly enhances the discoverability and reusability of a large, otherwise hard-to-access body of public health information in Canada.

Also in the Introduction (in the 1st paragraph) we have cited additional relevant administrative and historical publications that document Canadian notifiable disease surveillance programs; these references supply the additional context needed to interpret the data appropriately.

> **Reviewer**: *The paper lacks adequate coverage of zero reporting policies and reporting efficiency analyses.*

Reporting efficiency analysis is beyond the scope of the paper, but we do now discuss the issue in the 1st paragraph of the Limitations section. We emphasize that

we have provided information about which records represent true zeros (in Supporting Information S6). We also emphasize in the Explanation section (in the 2nd paragraph) and in the Data provenance section that we have provided data provenance information in our dataset that will allow users to reach their own conclusions about issues like reporting efficiency.

> **Reviewer**: *Provincial abbreviations need clarification; a map would be beneficial for readers.*

Thank you for this suggestion. We now provide a map (Figure 1), with abbreviations and full names of provinces and territories. This map also clarifies the grouping of provinces and territories into regions, which are used in the whooping cough example.

> **Reviewer**: *The figures have substantial presentation issues:*

We have addressed the concerns raised regarding the presentation of the figures, as we describe in our responses to the following issues. We have edited all figures to comply with PLoS style, and uploaded each figure separately as a set of tiff files.

> **Reviewer**: *Figure 1's caption is insufficient and the figure is difficult to interpret. Colors should be referenced rather than wavelengths, and the horizontal lines are unexplained.*

(Figure 1 is now Figures 2-4 in the revised manuscript)

We have clarified our description of the colour scale in the caption to give the colour for each time scale: “Colours indicate reporting frequency: weekly (blue), monthly (orange), and quarterly (red).”

We provide a detailed explanation of the horizontal lines, which we now believe are better thought of as ‘thin horizontal patterns’. In particular, we write “Thin horizontal patterns arise because provinces and territories differ in data availability and reporting frequency: when data are available for only some provinces, or when provinces and territories report at different time scales, the resulting variation appears as horizontal features due to the fixed top-to-bottom ordering of provinces and territories within each disease.”

This was a large multi-panel, multi-page figure designed to convey a substantial amount of information, spanning three full pages of high-resolution plots. To support clear interpretation, we have taken several steps to improve the presentation. Each panel is now its own figure (Figs 2-4) to comply with PLoS style. Each caption now highlights the multi-figure structure by providing links to the other two figures. The caption for Fig 2 gives the full description of the entire set of three plots. The captions for Figs 3 and 4 provide the following sentence: “*Please see the caption for Fig 2 for a full description of all of these plots.”* This full caption describes the horizontal patterns and other key features.

> **Reviewer**: *Figure 2 uses an inappropriate square root scale (a logarithmic scale would be more suitable) and should display absolute numbers rather than rates.*

(Figure 2 is now Figure 5 in the revised manuscript)

We used a square root scale because our incidence data include zeros, making a logarithmic scale not possible. The square root transformation has a similar visual effect, enhancing the visibility of smaller peaks in incidence that might otherwise be obscured on a linear scale.

We plot incidence rates per 100,000 to make incidence comparable across provinces and territories, and with other studies that follow this convention for reporting.

> **Reviewer**: *The inadequate horizontal scale in Figure 2 fails to identify the month of peak occurrence.*

(Figure 2 is now Figure 5 in the revised manuscript)

Thank you for this comment. Although the purpose of this figure is to show spatial synchrony, the month of peak occurrence is critical for interpreting our new results in the light of previous work in the U.S. We have added a figure in the Supplementary appendix that clearly shows that the peak timing was almost always between August and October in Canadian provinces. We reference this Supplementary figure both when describing the figure on spatial synchrony (1st paragraph in the Examples section) and when discussing the U.S. study (3rd paragraph in the Explanation section).

> **Reviewer**: *A more concise paper focusing on the history of disease notifications in Canada, accompanied by an announcement of the database's availability, with the current appendix material serving as supplementary information for interested researchers.*

We have renamed the “Appendix” as “Supplementary Information”. Consequently the main text is much shorter (2458 words).

As noted above, the introduction and Supplementary Information now include detailed references to historical federal and provincial data sources, including publication titles and time ranges, as well as relevant administrative and historical literature on Canadian notifiable disease surveillance programs. These additions support interpretation of our data set and improve the discoverability of additional hard-to-access historical data. The historical literature we cite already provides overviews of the evolution of disease notification systems in Canada.
