# Supplementary material for: A century of weekly notifiable disease incidence data by province in Canada
Source: PLOS Glob Public Health. 2025 Dec 17;5(12):e0005550. doi: 10.1371/journal.pgph.0005550 (PMC12711055; doi:10.1371/journal.pgph.0005550)
Supplement: Attachment — Submitted filename: PLOS GLOBAL PUBLIC HEALTH REVIEW.pdf [file pgph.0005550.s003.pdf]

This seems like a revised submission. Nevertheless, I commend the authors for the curation of such an important historical data. While an impressive and potentially time-consuming piece of work, there are some ambiguities that need clarification for a better understanding of what was done and to make the reporting clearer and more useful to researchers and other stakeholders. The PHAC, which I believe should have the entire curated data; albeit, potentially in typewritten/handwritten hard copies, as the authors suggested, provides summaries of the data from 1924-2022, but the authors have assembled incidence values from 1903-2021, which means an additional data from 21 years before 1924 and exclusion of 2022 data. It would be helpful to explain this disparity in the dates, particularly of the earliest available data because there is likely a reason why PHAC limited their data summaries to from 1924. There is also the need for the readers and potential users of the data to understand how the data differs from the summaries provided via PHAC because there are likely differences, however subtle they may be (for example, perhaps, PHAC provides intra-annual data summaries versus the sub-annual data, which is the focus of this work). It is worth mentioning that transparency, details, and full declaration are the key in data curation. These said, it would be helpful if the authors could also address the following concerns:

1. The terms intra-annual and sub-annual are of course timescales within a year but are nuanced and could create conflicts in the minds of some of the readers. As far as I am aware, intra-annual is a more precise term when describing events or variations in events happening within a year, while sub-annual is a broader term that encompasses any tracking of events less than a year. As such, it would be helpful to have these clarified at their first mention, and for consistency in their use throughout the study reporting.
2. The term sub-national needs to be clarified for less ambiguity. Perhaps, the authors are referring to provinces/territories, or maybe to regions, for example, the Prairies, Atlantic Canada etc.
3. The statement "*We developed open-source pipelines to harmonize these spreadsheets into CSV files that blend data across sources*" is unlikely to make sense to many, so needs clarifying/simplifying.
4. This study was conducted systematically, and therefore, could be likened to a systematic evidence review. As such, and for full transparency, the authors should consider adding more information to the methods to explain who (and how many) conducted literature/data search and how potential errors were mitigated, who (and how many) conducted data entry and what is meant by the statement "*We took an iterative approach to resolving unclear numbers by 'guessing and then refining our guesses' if necessary*", who (and how many) conducted data preparation, and the necessary quality checks/assessments that were made to mitigate errors.
5. The authors reported that "*the focus was on sub-annual and sub-national data and that removing redundancy yielded 934,010 weekly, monthly, or quarterly incidence values broken down by province/territory, containing 139 diseases*." It would be helpful to report what made an incidence value redundant, and why the focus was on sub-annual data that is clearly not truly representative of a year's incidence report rather than the more appropriate intra-annual data that covers incidence in a full year. Moreover, you cannot accurately explore variability in events in a year or describe seasonality of an

event, as the authors have claimed, without having considered the event incidence throughout a full year, in this case, intra-annual and not sub-annual data. Further, does 934,010 refer to week, month, or quarter? The sentence is muddled up, so needs clarifying.

6. Perhaps I am missing something, but I do not understand the statement "*Our sub-annual, sub-national data enable research on intra-annual and inter-provincial patterns in Canada and facilitate comparisons with U.S. data*". How can sub-annual inform intra-annual, when intra-annual encompasses a whole year (meaning that it includes sub-annual) whereas sub-annual includes just a portion of a year?
7. Although trivial, you do not start a sentence with year in digits – so, instead of "*2024 marked the 100th anniversary since Canada ……*", the authors should consider revising to "*The year 2024 marked the….*". Similar revision should be made wherever else such a reporting may have been made.
8. Finally, if at all useful, the authors could refer to this study on whooping cough from Manitoba (https://pubmed.ncbi.nlm.nih.gov/35045177/).
