# Supplementary material for: A century of weekly notifiable disease incidence data by province in Canada
Source: PLOS Glob Public Health. 2025 Dec 17;5(12):e0005550. doi: 10.1371/journal.pgph.0005550 (PMC12711055; doi:10.1371/journal.pgph.0005550)
Supplement: Attachment — Submitted filename: minor-revisions-reponse-plos-gph-for-plos-latex-template.pdf [file pgph.0005550.s004.pdf]

Thank you for the opportunity to revise our manuscript and for the thoughtful, constructive feedback. Our point-by-point responses follow below.

> **Reviewer #1:** *This manuscript presented a valuable work that shows the establishment of a comprehensive and accessible dataset of historical record of Canadian's notifiable disease incidence is greatly helpful for epidemiological studies. I do appreciate this hard work and acknowledge its value, and I think the comments from the previous reviewer have been properly responded in this revised version. I only have some very slight comments listed below.*

> **Reviewer #1:** *In my opinion, this manuscript is emphasizing the importance of setting up a comprehensive and high resolution dataset of notifiable disease incidence record, it might be better if the authors could indicate that from the title.*

Response: We have changed the title to "A Century of Weekly Notifiable Disease Incidence Data by Province in Canada".

> **Reviewer #1:** *In discussion section, future directions, the authors may also discuss how this dataset could help in interdisciplinary studies. For example, incorporate with economy and urbanization studies, how the change patterns of infectious disease incidence in Canada were related with the progress of urbanization, should also be worth studying.*

Response: Thank you for the suggestion. We have added a brief discussion of interdisciplinary research in the Conclusion (Paragraph 2).

> **Reviewer #1:** *At the end of subsection 3.4 Conclusion, instead of urging the government to require public health agencies to release higher resolution data as soon as possible, the authors might use a gentle wording to encourage the government to do that, and may also add up a bit more brief discussion about some potential obstacles that might hinder the public health agencies to release such data.*

Response: These agencies are already stretched to do more with less, and we have softened our language to acknowledge this challenge (Conclusion, Paragraph 3).

> **Reviewer #2:** *This seems like a revised submission. Nevertheless, I commend the authors for the curation of such an important historical data. While an impressive and potentially time-consuming piece of work, there are some ambiguities that need clarification for a better understanding of what was done and to make the reporting clearer and more useful to researchers and other stakeholders.*

> **Reviewer #2:** *The PHAC, which I believe should have the entire curated data; albeit, potentially in typewritten/handwritten hard copies, as the authors suggested, provides summaries of the data from 1924-2022, but the authors have assembled incidence values from 1903-2021, which means an additional data from 21 years before 1924 and exclusion of 2022 data. It would be helpful to explain this disparity in the dates,*

particularly of the earliest available data because there is likely a reason why PHAC limited their data summaries to from 1924.

Response: To avoid the impression that PHAC necessarily holds all historical notification data (they may, although this is unlikely), we revised the introduction to emphasize that Canadian disease surveillance has always been a multi-jurisdictional effort. We also clarify that our dataset integrates provincial publications, some predating the federal notification system established in 1924, as well as federal summaries and more recent records obtained directly from provincial agencies. By stating that 1924 marked the establishment of the federal notification system, we clarify why the data summaries provided online by PHAC (a federal agency) begin in 1924 (Introduction, top of Paragraph 3).

Reviewer #2: *There is also the need for the readers and potential users of the data to understand how the data differs from the summaries provided via PHAC because there are likely differences, however subtle they may be (for example, perhaps, PHAC provides intra-annual data summaries versus the sub-annual data, which is the focus of this work). It is worth mentioning that transparency, details, and full declaration are the key in data curation.*

Response: The PHAC portal data are at lower spatial and temporal resolution than our data – this is indeed the main purpose for our study. We have now clarified what we mean by sub-annual and sub-national in the introduction, in order to help make this point (Introduction, Paragraph 2).

With these terms clearly defined, we also clarify in the introduction that our approach is to surface the existing but hard-to-find sub-annual and sub-national data that the Canadian surveillance system has produced (Introduction, Paragraph 3). The Quality control sections in both the main text (Materials and methods, Quality control subsection) and Supporting Information (Section S1.5) discuss differences between the PHAC portal and the CANDID dataset. We briefly mention that the two sources are not perfectly consistent in the Data Entry section (Materials and methods, Data entry subsection). The appendix contains a graph illustrating discrepancies in chickenpox incidence between the two sources (Fig S5 in the Supporting Information). The manuscript website now contains similar graphs for all diseases shared by both datasets (https://github.com/canmod/candid/tree/main/output/phac-portal-comparisons) and we point to this URL in the Supporting Information (Caption for Figure S5).

Reviewer #2: *These said, it would be helpful if the authors could also address the following concerns:*

Reviewer #2: *The terms intra-annual and sub-annual are of course timescales within a year but are nuanced and could create conflicts in the minds of some of the readers. As far as I am aware, intra-annual is a more precise term when describing events or variations in events happening within a year, while sub-annual is a broader term that encompasses any tracking of events less than a year. As such, it would be helpful to have these clarified at their first mention, and for consistency in their use throughout the study reporting.*

Response: We have removed the terms ‘intra-annual’ and ‘inter-provincial’, and defined sub-annual and sub-national when they are first used in the Introduction (Paragraph 2). In the Abstract we give intuitive examples of sub-annual (weekly, monthly, quarterly) and sub-national (provincial, territorial) data. Instead of using intra-annual and inter-provincial variation, we describe in plain language the kind of variation that we have in mind: “seasonal patterns of incidence within years or … spatial patterns across provinces.” (Introduction, Paragraph 1).

> **Reviewer #2:** *The term sub-national needs to be clarified for less ambiguity. Perhaps, the authors are referring to provinces/territories, or maybe to regions, for example, the Prairies, Atlantic Canada etc.*

Response: Please see our response to the general comments above about the terms ‘sub-annual’ and ‘sub-national’. In particular, we focus on provincial and territorial data in our dataset, which we make clear in the Introduction (end of Paragraph 3).

> **Reviewer #2:** *The statement “We developed open-source pipelines to harmonize these spreadsheets into CSV files that blend data across sources” is unlikely to make sense to many, so needs clarifying/simplifying.*

Response: We agree, and thank you for this suggestion. We have heavily edited Paragraph 2 of the Abstract to use plain language and removed the technical terms ‘open-source’, ‘pipelines’, and ‘harmonize’.

> **Reviewer #2:** *This study was conducted systematically, and therefore, could be likened to a systematic evidence review. As such, and for full transparency, the authors should consider adding more information to the methods to explain who (and how many) conducted literature/data search and how potential errors were mitigated, who (and how many) conducted data entry and what is meant by the statement “We took an iterative approach to resolving unclear numbers by ‘guessing and then refining our guesses’ if necessary”, who (and how many) conducted data preparation, and the necessary quality checks/assessments that were made to mitigate errors.*

Response: We apologize for not making sufficiently clear how carefully our methodology was designed and implemented. The Supporting Information contains extensive details on our literature search, data entry, data preparation, and quality control efforts, including an entirely new section called Data Entry in the Supporting Information (Section S1.2). We have further augmented this Supporting Information to identify the team members who were involved in each of these project components (Section S1.1, Paragraph 5; Section S1.1, named paragraph *Provincial and territorial government data requests*; Section S1.2, Paragraph 3; Section S1.4, Paragraph 3; Section S1.5, Paragraph 1). We replaced the statement about “guessing and then refining …” with a longer and clearer explanation in the Data Entry section of the Methods (Data entry subsection). In the Supporting Information section titled “External Links”, we note that the following GitHub page provides download links for the CANDID data and includes additional technical detail that we considered too extensive for the appendix: https://github.com/canmod/iidda/blob/main/README.md.

> **Reviewer #2:** *The authors reported that “the focus was on sub-annual and sub-national data and that removing redundancy yielded 934,010 weekly, monthly, or quarterly*

incidence values broken down by province/territory, containing 139 diseases." It would be helpful to report what made an incidence value redundant, and why the focus was on sub-annual data that is clearly not truly representative of a year's incidence report rather than the more appropriate intra-annual data that covers incidence in a full year. Moreover, you cannot accurately explore variability in events in a year or describe seasonality of an event, as the authors have claimed, without having considered the event incidence throughout a full year, in this case, intra-annual and not sub-annual data. Further, does 934,010 refer to week, month, or quarter? The sentence is muddled up, so needs clarifying.

Response: Thank you for these comments. To clarify, we use sub-annual to refer to data reported at finer temporal resolution than one year (e.g., weekly, monthly, quarterly). Such series usually span full years – the term describes resolution, not partial coverage – and therefore make it possible to study variation within years (seasonal patterns) that cannot be examined using annual summaries. We have clarified this definition at its first use (Introduction, Paragraph 2). As noted above, we have also removed the terms intra-annual and inter-provincial to avoid confusion, instead describing directly the seasonal and spatial patterns that the data allow us to study (Introduction, Paragraph 1).

We no longer use the word 'redundant', and prefer the more neutral adjective 'overlapping'. We have now described in more detail what makes an incidence value redundant (without using that word) in the Abstract (Paragraph 3). This paragraph also specifies the exact numbers of incidence values associated with each sub-annual timescale.

Reviewer #2: *Perhaps I am missing something, but I do not understand the statement "Our sub-annual, sub-national data enable research on intra-annual and inter-provincial patterns in Canada and facilitate comparisons with U.S. data". How can sub-annual inform intra-annual, when intra-annual encompasses a whole year (meaning that it includes sub-annual) whereas sub-annual includes just a portion of a year?*

Response: Thank you for raising this point. To clarify, sub-annual refers to the temporal resolution of reporting (weekly, monthly, quarterly), not to partial coverage of a year. Sub-annual data typically extend across full years and therefore enable the study of variation within years. To prevent confusion, we have clarified the definition of sub-annual at first use and removed the terms intra-annual and inter-provincial, replacing them with plain descriptions such as "seasonal patterns within years" and "spatial variation across provinces." The revised sentence now reads: "Our sub-annual, sub-national data enable research on patterns of variation within years and across provinces in Canada, and facilitate comparisons with U.S. data." (Discussion, Paragraph 1).

Reviewer #2: *Although trivial, you do not start a sentence with year in digits – so, instead of "2024 marked the 100th anniversary since Canada ……", the authors should consider revising to "The year 2024 marked the….". Similar revision should be made wherever else such a reporting may have been made.*

Response: Agreed and implemented (Introduction, Paragraph 1).

> **Reviewer #2:** *Finally, if at all useful, the authors could refer to this study on whooping cough from Manitoba (https://pubmed.ncbi.nlm.nih.gov/35045177/).*

Response: We are grateful to the reviewer for pointing out this study, which supplements time-series of incidence with age-standardized incidence rates to improve estimates of vaccine efficacy against pertussis (whooping cough). Indeed this study was useful to further motivate some of our future directions: "Age data, for example, can be critical for estimating the impact of vaccination programmes in childhood diseases (e.g., [32])" (Discussion, Future directions subsection).

> **Reviewer #3:** *This is an interesting study detailing the creation of a dataset (CANDID) that can be used for epidemiology and public health. Below, I provide some comments that can help in strengthening the manuscript.*

> **Reviewer #3:** *Could the authors state and clarify the objectives of the study? From the introduction, it seems that the purpose of the study was to create a dataset that records the incidence of diseases in Canada over time.*

Response: Yes this is most of our objective. The other part is that we focus on sub-annual (weekly, monthly, quarterly) and sub-national (provincial, territorial) data because most annual and national data are already easy to access through PHAC's portal. We have now explicitly clarified this objective with the following sentence in the introduction: "Our objective is to make these sub-annual and sub-national disease incidence data, which have been difficult to obtain, conveniently available for public health and research use." (Introduction, Paragraph 3). In the abstract we state this same objective in the following sentence: "We digitized and collated these sources to make sub-annual and sub-national Canadian disease incidence data conveniently available for researchers." We have also modified the title so that it describes the major output of our work towards this objective: "A Century of Weekly Notifiable Disease Incidence Data by Province in Canada"

> **Reviewer #3:** *Since the manuscript has been sent to a global health journal, could the authors mention how this methodology can be applied to international settings, where most of the data is in hard copies?*

Response: We have added the following: "The process of assembling CANDID highlighted logistical obstacles that are likely to arise in other countries seeking to build comparable historical surveillance datasets" (Discussion, Logistical challenges subsection, Paragraph 1) and "By documenting our workflows and tools openly, we aim for CANDID to serve as both a resource and a practical starting point for researchers undertaking similar digitization efforts elsewhere." (Discussion, Logistical challenges subsection, Paragraph 4).

> **Reviewer #3:** *The authors have mentioned the limitations of the study; however, it will be worthwhile to state the logistical and other difficulties faced during the collection of data. This would be helpful for researchers in other countries if they want to create similar datasets.*

Response: Thank you for this excellent suggestion. Our Supporting Information details many of the logistical challenges that we faced, which were many. We have now added a Logistical

Challenges sub-section (Discussion, Logistical challenges subsection) in the discussion that we use to summarize these challenges and provide pointers to parts of the Supporting Information for extensive details.
